# A Hybrid Spectrum Access Strategy with Channel Bonding and Classified Secondary User Mechanism in Multichannel Cognitive Radio Networks

**DOI:** 10.3390/s19204398

**Published:** 2019-10-11

**Authors:** Yuan Zhao, Minglei Peng, Jiemin Liu

**Affiliations:** School of Computer and Communication Engineering, Northeastern University at Qinhuangdao, Qinhuangdao 066004, China; baimax9631@163.com (M.P.); liujm@neuq.edu.cn (J.L.)

**Keywords:** cognitive radio networks, multichannel, hybrid access mode, channel bonding, classified secondary users

## Abstract

Cognitive radio networks (CRNs) can improve spectrum utilization by allowing secondary users (SUs) to dynamically access channels unoccupied by primary users (PUs). The spectrum access strategy, as a point to enhance user performance, has received much attention. In this paper, we propose a hybrid access mode for network users in multichannel CRNs. For meeting different SU demands, SUs are classified as SU1s and SU2s. We further introduce a channel bonding scheme for high-priority (PU and SU1) user packets to enhance transmission efficiency. At the same time, we propose a hybrid spectrum access strategy for SU2 packets to improve their transmission stability. By establishing a Markov chain model, some important SU2 packets’ performance measures are derived. Furthermore, we display the comparison of hybrid, overlay and underlay modes by numerical results to analyze the advantages of different modes.

## 1. Introduction

With the wide application of wireless networks, network spectrum resources have become relatively scarce. However, in a traditional fixed-spectrum allocation mode, some of the spectrum is wasted by very poor utilization users [1]. As a promising technique, the cognitive radio network (CRN) [2] is perceived as an effective way to improve spectrum utilization. In CRNs, primary users (PUs) share their licensed spectra with secondary users (SUs) under the condition that the caused interference to the PUs does not reach to an unreasonable level.

In general, SUs have three spectrum access modes [3]—(1) Overlay mode, under which SUs are only allowed to access the spectrum that is not being used by PUs and SU transmission is interrupted by PUs; (2) Underlay mode: In this access mode, SUs are allowed to be transmitted with PUs by maintaining the interference level below a prescribed interference-threshold limit (ITL), such as transmit power, to ensure PU Quality of Service (QoS). Just as indicated in Reference [3], some feedback mechanisms may be used to periodically update the SU transmitters with the value of instantaneous interference generated on the PU receivers; (3) Hybrid mode, with which SUs first detect the state of the spectrum and then choose an appropriate spectrum access mode based on the spectrum-detecting results. We note that SUs have a spectrum-sensing ability in CRNs and this is an essential component of a CRN system [4]. It refers to detecting idle channels that can be utilized via the overlay mode or channels that can be utilized by underlay mode [5]. In hybrid access mode, if the spectrum is sensed to be occupied by PUs, the SUs choose the spectrum underlay access mode and transmit with ITL. Otherwise, SUs choose the spectrum overlay access mode and transmit with a higher rate.

In recent years, many researchers began to focus on analyzing system performance under hybrid access mode in CRNs to demonstrate its advantages. Oh et al. [6] proposed a hybrid mode where the transmitting SU packets could dynamically change from overlay mode to underlay mode to improve the throughput and transmission stability of SUs. In Reference [7], SUs were able to access the spectrum with a switching probability when PU transmission was detected. Wang et al. [8] considered a hybrid access mode that provided QoS guarantee for SUs. Ma et al. [9] studied the effect of incorrect spectrum sensing on CRN performance in hybrid mode. Liu et al. [10] established a queuing-theory model for a hybrid spectrum-sharing system and studied the system’s optimal gain strategy. They all showed the advantages of hybrid modes by comparing them with traditional transmission modes. However, there are few studies on hybrid modes for classified SU systems.

With the increase of mobile-network devices, there are various types of data with different QoS requirements. Therefore, it is necessary to classify SUs to meet different SU service demands. In recent years, some scholars began to focus on researching classified SUs in CRNs. In these studies, SUs were further divided into several categories with different priorities. Lee et al. [11] separated SUs into SU2 and SU1. They assumed that SU2 had lower priority than SU1. By establishing a continuous Markov chain, they analyzed SU2 and SU1 performance in a multichannel CRN. Zhang et al. [12] considered the transmission delay in a priority-based SU system. By employing a pre-emptive resume priority M/M/1 queuing model, they analyzed the influence of an interrupted packet-precedence scheme on performance measures. Results showed the QoS of the high-priority SUs could be effectively guaranteed. Zhao et al. [13] considered CRNs with prioritized SUs. By setting a dynamic access threshold to control SU2 packet access, they analyzed the influence of the access threshold on some performance measures, such as the throughput, delay and blocking rate of SU2. The classified SU system obtained several categories of SUs with different performance.

In a multichannel system, different packets can transmit in parallel on different channels. This parallel-transmission way provides the possibility to increase the performance of network users. Some scholars introduced hybrid access mode into the system-performance analysis of multichannel systems. Zou et al. [14] introduced a hybrid multichannel cognitive radio system with ideal spectrum perception and estimation. SUs adapted the access mode by the detected status of the PUs. Huo et al. [15] proposed a hybrid mode in a multichannel network where SU packets could be dynamically switched between overlay and underlay modes based on PU existence. The transmitting SU packets in underlay mode would look for available channels that were not occupied by another packet to switch to overlay mode.

In addition, the channel bonding mechanism could bind multiple channels in a continuous spectrum into one that could obtain larger bandwidth [16]. Unlike carrier aggregation (CA), it could link multiple discrete segments of the spectrum [17]. The channel bonding mechanism has been widely used in various traditional networks [16]. The advantage of using the channel bonding mechanism in CRNs is that it provides greater bandwidth, lower networking complexity and greater channel processing power [18]. Rehmani et al. [19] studied the application of the channel bonding mechanism in a CRN environment. They assumed that there were multiple channels in the network and the control node perceived the channel user’s situation and could bind multiple channels for different medical devices. Through the application of this scenario, they discussed how the general technique of channel bonding could be applied to other, different scenarios. Katayama et al. [20] considered a channel bonding mechanism in cognitive wireless networks. The licensed spectrum was divided into multiple channels and all channels were bonded when authorized users transmitted. When a cognitive user used it, the bonding channel was bonded based on the number of available channels.

To our best knowledge, most studies of hybrid access mode have not considered the classified SU mechanism. Considering the different QoS demands of SUs, it is meaningful to classify SUs for wider application. The channel bonding strategy, as a widely used technique in traditional networks, is also rarely considered in CRNs. Therefore, in this paper we present a hybrid spectrum access strategy with classified SUs and a channel bonding mechanism in multichannel CRNs. Moreover, we note that a Markov process in mathematics is defined as a stochastic process. In a Markov process, possible states are finite and are denoted with non-negative integers, which are suitable to represent packet numbers and channel states in CRNs. Just as described in the survey of Reference [21], the Markov chain model is widely used in CRN analysis. For the hybrid access mode proposed in this paper, system states are finite and we could explicitly obtain the state-transition matrix with Markov chain model analysis. Therefore, we analyzed system performance by using the Markov chain model approach in this paper. The main contributions of this paper are summarized as follows: 

(1) We proposed a hybrid access mode with channel bonding mechanism in CRNs with classified SUs.

(2) We built and analyzed a Markov chain model, which is well-suited for the performance analysis of CR model. With steady-state analysis, we obtained some important performance measures.

(3) We compared the proposed hybrid mode with the traditional overlay and underlay modes to show the advantage of different spectrum access strategies.

The paper is organized as follows. A system model for the hybrid access strategy with channel bonding and a classified SU mechanism is presented in Section 2. Some important performance indicators, such as the blocking rate, throughput and average delay of SU2 packets are demonstrated in Section 3. In Section 4, we discuss performance measures with numerical experiments. We compare the proposed hybrid mode with traditional underlay and overlay modes in two different situations. Finally, a conclusion is given in Section 5.

## 2. System Model

### 2.1. Model Assumption

We assumed there was one PU, one SU1 and one SU2 in the system and we focused on the system actions of different packets generated from the PU, SU1 and SU2. We supposed that the spectrum consisted of *M* channels and each channel had the same transmission capability. Generally, each channel is available for one packet transmission. With channel bonding, multiple channels can be aggregated into a single channel with larger bandwidth for one packet transmission. PU packets have the highest priority and the transmission of the PU packets therefore applied the channel bonding mechanism of this paper.

SU packets have two categories, namely, SU1 (with higher priority) and SU2 packets. SU1 packets adopt the overlay access mode with the channel bonding mechanism. Therefore, transmitting SU1 packets use a bonding channel (aggregated by all channels) as PU packets but their transmission can be interrupted by newly arrived PU packets. SU2 packets employ hybrid access mode without a channel bonding mechanism, so a transmitting SU2 packet only occupies a single channel and its transmission changes dynamically based on the state of the system. Considering the different transmission demands, a limited buffer was set to accept the SU2 packets and the buffer capacity was *K*.

**Figure 1:**
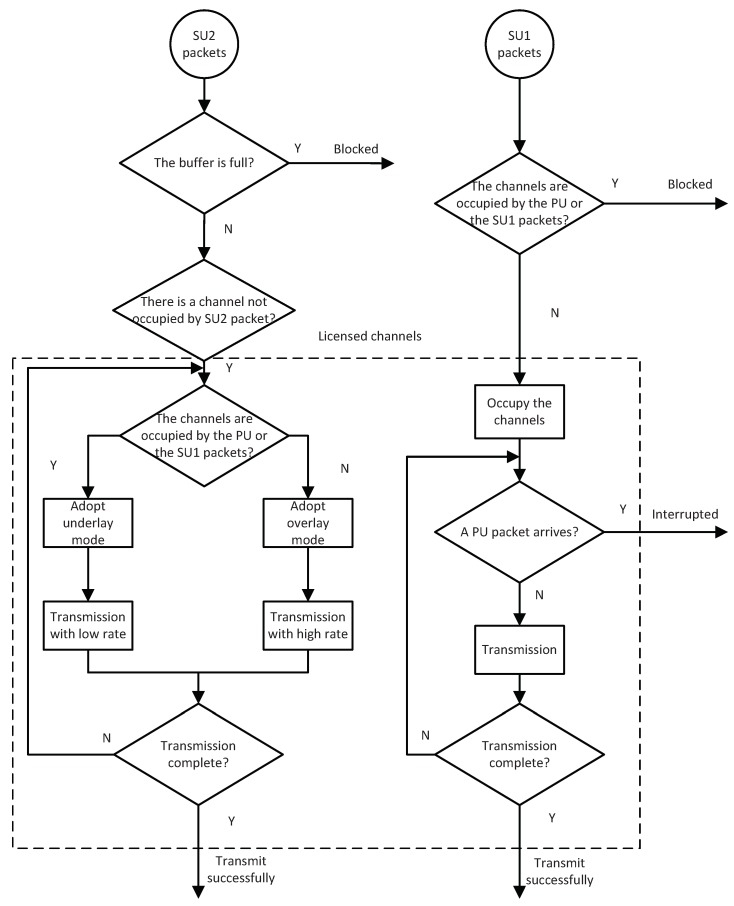
System behavior of SU1 and SU2 packets.

Figure 1 more clearly and intuitively shows the system behavior of the SU1 and SU2 packets.

When the system detects the arrival of a PU packet, if the bonding channel has been used by another PU packet, the system refuses the arrival of the new PU packet. Otherwise, the new PU packet uses the bonding channel. A newly arrived SU1 packet could occupy channels only when the bonding channel is not being used by a PU or an SU1 packet (ideal state). If the system has already accepted another PU or SU1 packet (busy state), the system blocks a newly arrived SU1 packet. A newly arrived PU packet also interrupts the transmission of the SU1 packet in the bonding channel. The interrupted SU1 packet leaves and seeks another available spectrum.

The SU2’s buffer controls the access of the newly arrived SU2 packets. If the buffer is full, the system blocks a newly arrived SU2 packet. Otherwise, the newly arrived SU2 packet enters the buffer and waits for an available channel to transmit. SU2 packets can coexist with PU or the SU1 packets in the channels, and each channel can be used by an SU2 packet. SU2 packets in the buffer continue seeking an available channel that is not used by another SU2 packet to enter. The transmission mode of SU2 packets in the channels depends on the system state. If the bonding channel is not occupied by a PU or an SU1 packet, SU2 packets select overlay mode and transmit with a high rate. Otherwise, SU2 packets work in underlay mode and transmit with a low rate.

Based on the model assumption mentioned above, the transmission of PU and SU1 packets is not affected by SU2 packets. They are similar to a traditional overlay mode without a classified SU mechanism. For SU2 packets, hybrid access mode can improve the stability of SU2 packet transmission and each SU2 packet occupying one channel allows the system to simultaneously handle as much as SU2 packets. So, we assumed two types of SU packets with different priorities to meet the different demands of SUs. System performance could be analyzed by establishing a discrete-time Markov chain model.

In the system, we assumed that time was divided into equal segments and we denoted the slot boundary as t=1,2,⋯. Each type of packet is respectively considered as a whole. The arrival intervals of the SU1, SU2 and PU packets were assumed to follow geometric distributions with arrival rates $\lambda_{21}\left({\bar{\lambda }}_{21}=1-\lambda_{21},0<\lambda_{21}<1\right)$, $\lambda_{22}\left({\bar{\lambda }}_{22}=1-\lambda_{22},\;0<\lambda_{22}<1\right)$, and $\lambda_{1}\left({\bar{\lambda }}_{1}=1-\lambda_{1},\;0<\lambda_{1}<1\right)$. The transmission times of the SU1, SU2 and PU packets were assumed to follow geometric distributions with service rates $\mu_{21}\left({\bar{\mu }}_{21}=1-\mu_{21},0<\mu_{21}<1\right)$, $\mu_{22}\left({\bar{\mu }}_{22}=1-\mu_{22},0<\mu_{22}<1\right)$ (in underlay mode $\mu_{22}=\mu_{H}$, in overlay mode $\mu_{22}=\mu_{L}$ and $\mu_{L}<\mu_{H}$) and $\mu_{1}\left({\bar{\mu }}_{1}=1-\mu_{1},0<\mu_{1}<1\right)$.

Let Sn be the SU2 packet number in the buffer and channels at moment $t=n^{+}$. Let Ln be the PU and the SU1 packet channel-occupying condition at moment $t=n^{+}$. Ln=0 means there are no PU or SU1 packets in the system to occupy the channels, Ln=1 represents that channels are occupied by an SU1 packet and Ln=2 means that a PU packet occupies the channels. Then, we noted that {Sn,Ln} constituted a two-dimensional discrete-time Markov chain. With the model conception, we could give state space Ω of {Sn,Ln} as follows:

(1) Ω={(i,j):0≤i≤M+K,j=0,1,2}.

### 2.2. Model Analysis

**Figure 2:**
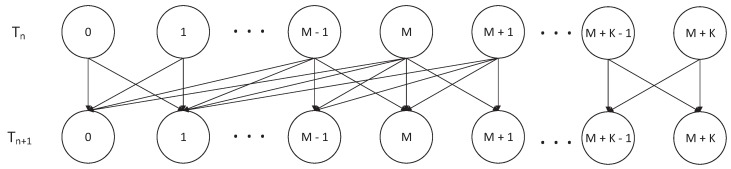
Markov state-transition diagram of number of SU2 packets.

We noted that Sn in Markov chain {Sn,Ln} denoted SU2 packet number in the buffer and channels. According to the hybrid access mode in this paper, we could give the Markov state-transition diagram of the number of SU2 packets in Figure 2. In Figure 2, $T_{n}$ represents a possible initial moment and $T_{n+1}$ represents the next moment.

We marked P as the state-transition probability matrix of {Sn,Ln}. According to the Markov state-transition diagram in Figure 2, the form of P could be demonstrated as follows:

(2) $$P=\left(\begin{array}{cccccc} P_{0,0} & P_{0,1}\\ P_{1,0} & P_{1,1} & P_{1,2}\\ \vdots & \vdots & \ddots \\ P_{M,0} & P_{M,1} & \cdots & P_{M,M+1}\\ \; & P_{M+1,1} & P_{M+1,2} & \cdots & P_{M+1,M+2}\\ \; & \ddots & \ddots & \; & \ddots \\ \; & \; & P_{M+K-1,K-1} & P_{M+K-1,K} & \cdots & P_{M+K-1,M+K}\\ \; & \; & \; & P_{M+K,K} & \cdots & P_{M+K,M+K} \end{array}\right).$$

$P_{i,j}$ is the transition-probability sub-block when the total SU2 packet number in the buffer and channels transfers from *i* (i=0,1,⋯,M+K) to *j* (j=0,1,⋯,M+K).

**Figure 3:**
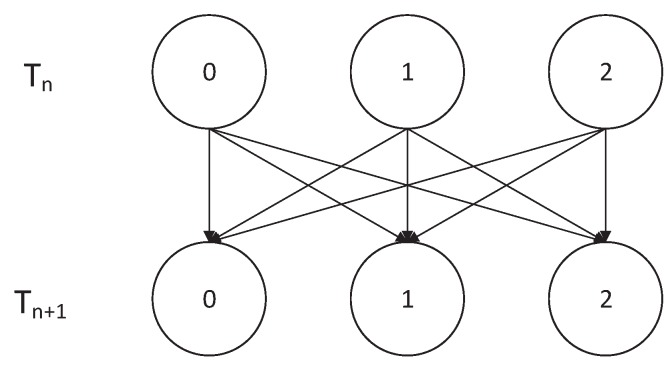
Markov state-transition diagram for primary user (PU) and the secondary user (SU1) packets’ channel-occupying condition.

Based on the transmission mechanism of hybrid access mode, the transmission of SU2 packets does not affect the transmission of SU1 and PU packets. We could ignore SU2 packets when analyzing the state change of the SU1 and the PU packets. We noted that Ln in Markov chain {Sn,Ln} denoted the PU and the SU1 packets’ channel-occupying condition and the value of Ln could be 0, 1, or 2. Based on the hybrid access mode in this paper, we could give the Markov state-transition diagram for the PU and the SU1 packets’ channel-occupying condition in Figure 3. In Figure 3, $T_{n}$ represents a possible initial moment and $T_{n+1}$ represents the next moment.

Let matrix A be the state-transition probability of Ln. $A_{u,v}$ in *A* is the transition probability of Ln from *u* (u=0,1,2) to *v* (v=0,1,2). According to the Markov state-transition diagram in Figure 3, *A* can be given as follows:

(3) $$A=\left(\begin{array}{ccc} {\bar{\lambda }}_{1}{\bar{\lambda }}_{21} & {\bar{\lambda }}_{1}\lambda_{21} & \lambda_{1}\\ {\bar{\lambda }}_{1}{\bar{\lambda }}_{21}\mu_{21} & {\bar{\lambda }}_{1}\left({\bar{\lambda }}_{21}{\bar{\mu }}_{21}+\lambda_{21}\right) & \lambda_{1}\\ {\bar{\lambda }}_{1}{\bar{\lambda }}_{21}\mu_{1} & {\bar{\lambda }}_{1}\lambda_{21}\mu_{1} & {\bar{\lambda }}_{1}{\bar{\mu }}_{1}+\lambda_{1} \end{array}\right).$$

In hybrid access mode, the transmission rate of SU2 packets is influenced by the system state. When Ln=0, the system is in idle state, the SU2 packet transmission rate is $\mu_{22}=\mu_{H}$. When Ln=1 or Ln=2, the system is in a busy state and SU2 packet transmission rate $\mu_{22}=\mu_{L}$. $B_{m,n}$ denotes the state-transition probability for the number of SU2 packets changing from *m* to *n*. *m* and *n* denote the number of SU2 packets in one time point and the next time point, respectively. For example, $B_{1,2}$ denotes the probability that the number of SU2 packets changes from 1 to 2.

(1) When m=0, there are no SU2 packets in the system. The value of *n* can only be 0 and m+1.

(4) $$B_{m,n}=\left\{\begin{array}{cc} {\bar{\lambda }}_{22}, & n=0\\ \lambda_{22}, & n=m+1. \end{array}\right.$$

(2) When 1≤m≤M, there are *m* SU2 packets in the channels but zero SU2 packets in the buffer. The range of *n* is [0,m+1].

(5) $$B_{m,n}=\left\{\begin{array}{cc} {\bar{\lambda }}_{22}\mu_{22}^{m}, & n=0\\ {\bar{\lambda }}_{22}C_{m}^{m-n}\mu_{22}^{m-n}{\bar{\mu }}_{22}^{n}+\lambda_{22}C_{m}^{m-n+1}\mu_{22}^{m-n+1}{\bar{\mu }}_{22}^{n-1}, & 1\leq n\leq m\\ \lambda_{22}{\bar{\mu }}_{22}^{m}, & n=m+1. \end{array}\right.$$

(3) When M≤m≤M+K−1, channels are filled up by SU2 packets but the buffer is not full. The range of *n* is [m−M,m+1].

(6) $$B_{m,n}=\left\{\begin{array}{cc} {\bar{\lambda }}_{22}\mu_{22}^{M}, & n=m-M\\ {\bar{\lambda }}_{22}C_{M}^{m-n}\mu_{22}^{m-n}{\bar{\mu }}_{22}^{M-m+n}+\lambda_{22}C_{M}^{m-n+1}\mu_{22}^{m-n+1}{\bar{\mu }}_{22}^{M-m+n-1}, & m-M+1\leq n\leq m\\ \lambda_{22}{\bar{\mu }}_{22}^{M}, & n=m+1. \end{array}\right.$$

(4) When m=M+K, the buffer and channels are both filled up by SU2 packets. If a new SU2 packet arrives and none of the SU2 packets in the channels is successfully transmitted, the newly arrived packet is blocked. The range of *n* is [m−M,m].

(7) $$B_{m,n}=\left\{\begin{array}{cc} {\bar{\lambda }}_{22}\mu_{22}^{M}, & n=m-M\\ {\bar{\lambda }}_{22}C_{M}^{m-n}\mu_{22}^{m-n}{\bar{\mu }}_{22}^{M-m+n}+\lambda_{22}C_{M}^{m-n+1}\mu_{22}^{m-n+1}{\bar{\mu }}_{22}^{M-m+n-1}, & m-M+1\leq n\leq m-1\\ {\bar{\mu }}_{22}^{M}+\lambda_{22}C_{M}^{1}\mu_{22}{\bar{\mu }}_{22}^{M-1}, & n=m. \end{array}\right.$$

By substituting $\mu_{22}$ with different transmission rates ($\mu_{H}$ and $\mu_{L}$), we can get the SU2 packets’ transition-probability matrix in underlay and overlay mode, respectively. When $\mu_{22}=\mu_{L}$, we denoted *B* as BL and BL represents the transition-probability matrix of SU2 in underlay mode. When $\mu_{22}=\mu_{H}$, we denoted *B* as BH and BH represents the transition-probability matrix of SU2 in overlay mode.

Through the above transition-probability matrix *A*, BL and BH, we can give each $P_{i,j}$ in *P* as follows:

(8) $$P_{i,j}=\left(\begin{array}{ccc} A_{0,0}BH_{i,j} & A_{0,1}BH_{i,j} & A_{0,2}BH_{i,j}\\ A_{1,0}BL_{i,j} & A_{1,1}BL_{i,j} & A_{1,2}BL_{i,j}\\ A_{2,0}BL_{i,j} & A_{2,1}BL_{i,j} & A_{2,2}BL_{i,j} \end{array}\right).$$

Until now, each transition-probability sub-block in *P* was presented. Let $\pi_{i,j}$ be the steady-state distribution of Markov chain {Sn,Ln}. Based on the framework of matrix *P*, $\pi_{i,j}$ is defined as follows:

(9) $$\pi_{i,j}=\lim_{n\to \infty }P\left\{Sn=i,Ln=j\right\}.$$

We marked Π as steady-state probability vector $\Pi =\{\pi_{0,0},\pi_{0,1},\pi_{0,2},\pi_{1,0},\cdots ,\pi_{M+K,2}\}$. By solving ΠP=Π and Πe=1 [22], we could obtain the result of Π. By referencing the numerical calculation method in Reference [23], we give the steps to derive Π as follows:

**Step 1.** Set $E={\left(\begin{array}{ccc} 1\\ & \ddots \\ & & 1 \end{array}\right)}_{\left(3M+3K+3)\times (3M+3K+3\right)}$ and $e={\left(\begin{array}{c} 1\\ \vdots \\ 1 \end{array}\right)}_{(3M+3K+3)\times 1}$.

**Step 2.** Construct $D=\left(P-E,e\right)$.

**Step 3.** Set d=${\left(\begin{array}{c} 0,0,\cdots ,0,1 \end{array}\right)}_{1\times (3M+3K+4)}$.

**Step 4.** Resolve Z=d/D

**Step 5.** Return Π=Z.

## 3. Performance Measures

In this part, we give the definitions and expressions of some significant performance measures. In the hybrid mode with classified SUs proposed in this paper, the system actions of PU and SU1 packets were not influenced by SU2 packets and the performance measures of PU and SU1 packets were the same as those in traditional overlay mode, which have been extensively studied. Therefore, in this paper we only focused on the performance of SU2 packets.

First, we counted the blocked SU2 packets’ number per slot. This could be defined as blocking rate β of the SU2 packets. When all channels are occupied and the buffer is fully occupied by SU2 packets, a newly arrived SU2 packet is blocked. The expression of blocking rate β can be given as follows:

(10) $$\beta =\lambda_{22}\left(\pi_{M+K,0}{\bar{\mu }}_{H}^{M}+\left(\pi_{M+K,1}+\pi_{M+K,2}\right){\bar{\mu }}_{L}^{M}\right).$$

Next, we considered the number of SU2 packets successfully transmitted per slot. This could be defined as throughput θ. Since the transmission of SU2 packets would not be interrupted to loss in the proposed hybrid access mode, any SU2 packet that enters the system without being blocked is successfully transmitted. The expression of throughput θ is given as follows:

(11) $$\theta =\lambda_{22}-\beta .$$

Then, we defined the average time of an SU2 packet staying in the system as average delay δ of SU2 packets. The expression of average delay δ is derived as:

(12) $$\delta =\frac{E(SU2)}{\theta }$$

where E(SU2) represents the average SU2 packet number in the buffer and channels. E(SU2) is given as follows:

(13) $$E\left(SU2\right)=\sum_{i=0}^{M+K}i\left(\pi_{i,0}+\pi_{i,1}+\pi_{i,2}\right).$$

## 4. Numerical Experiments

### 4.1. Change Trends of SU2 Performance Measures

In this part, we discuss the change trends of SU2 performance measures. In the numerical results, we mainly focused on the influence of four parameters. First, channel number *M* influences the system-transmission capacity to all kinds of users. Second, the arrival rates of the SU1 and PU packets ($\lambda_{21}$ and $\lambda_{1}$) affect the channel state to the SU2 packets. Finally, buffer capacity *K* decides how many SU2 packets are to be accepted by the system. Moreover, the other system parameters were set as follows: $\lambda_{22}=0.4,\mu_{H}=0.15,\mu_{L}=0.03$.

**Figure 4:**
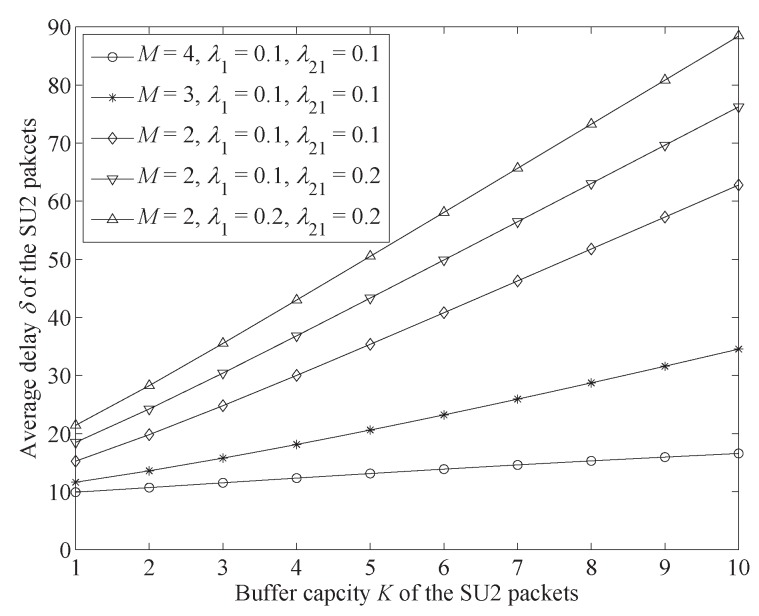
Average delay δ change trends with increase of buffer capacity *K* of SU2 packets under different conditions ($\lambda_{22}=0.4,\mu_{H}=0.15,\mu_{L}=0.03,\mu_{1}=\mu_{21}=M\left(\mu_{H}-\mu_{L}\right)$).

Figure 4 shows the change trends of SU2 packet average delay δ.

From Figure 4, it can be seen that the average delay becomes higher as the buffer capacity increases. This is because a larger buffer capacity can hold more SU2 packets, which lengthens the SU2 packets’ waiting time in the buffer. In addition, we also found that the rise in the arrival rate of SU1 and PU packets increased SU2 packets’ average delay. The reason is that SU1 and PU packets occupy channels more frequently, which leads to SU2 packets staying longer in underlay mode with a lower transmission rate. Moreover, we could find that the increase in the number of channels reduces average delay. This is because this increase does not only enhance the SU2 packets’ transmission rate but also increases PU and SU1 packet transmission rates. This gives SU2 packets a longer time in overlay mode with a higher transmission rate.

**Figure 5:**
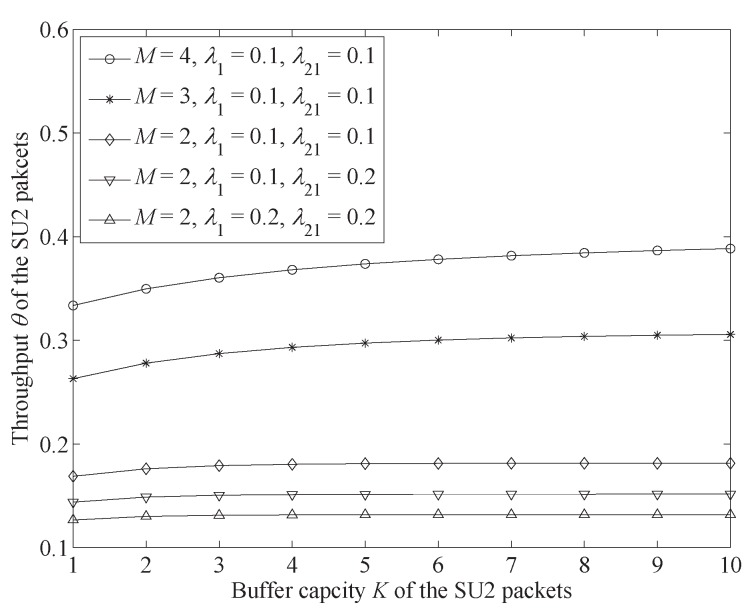
Throughput θ change trends with the increase of buffer capacity *K* of SU2 packets under different conditions ($\lambda_{22}=0.4,\mu_{H}=0.15,\mu_{L}=0.03,\mu_{1}=\mu_{21}=M\left(\mu_{H}-\mu_{L}\right)$).

Figure 5 shows the change trends of SU2 packet throughput θ.

Figure 5 shows that a greater buffer capacity increases throughput. This is because a larger buffer capacity reduces the blocking rate. However, this trend becomes less noticeable when the number of channels is decreased. This is because the most important factor for restricting throughput is average transmission rate. When throughput is close to the average transmission rate, the increase in buffer capacity has little improvement in throughput. We also found that the reduction of channel number and the increase of PU and SU1 packets’ arrival rates decreases SU2 packet throughput. This is because a higher PU or SU1 packet arrival rate increases underlay mode time of SU2 packets and a smaller number of channels reduce the transmission capacity of the system. Both of these two results reduce the throughput of the SU2 packets.

Figure 4 and Figure 5 show that buffer capacity has an important influence on SU2 packet performance. An increase in buffer capacity could improve throughput but prolong average delay. Therefore, we aimed to set buffer capacity according to different network requirements. For example, for a system with a higher need for throughput, we could increase buffer capacity. On the contrary, we may have needed to decrease the buffer capacity for a system with lower tolerance for delay.

### 4.2. Numerical Result Comparison between Three Modes

Here, we compare the proposed hybrid mode with the traditional underlay and overlay modes to analyze their advantages on SU2 packet performance.

**Figure 6:**
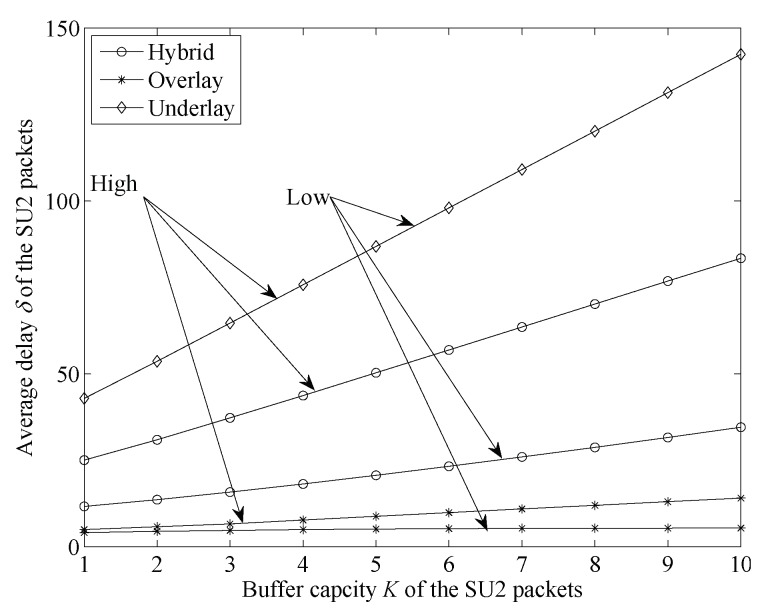
Average delay change trends comparison between three access modes under different high-priority user arrival rates ($M=3,\lambda_{22}=0.4,\mu_{H}=0.15,\mu_{L}=0.03,\mu_{1}=\mu_{21}=M\left(\mu_{H}-\mu_{L}\right)$).

Figure 6 shows the average delay between the three modes with two different high-priority user-arrival-rate settings.

From Figure 6 we can see that, in hybrid and overlay modes, the change of high-priority user arrival rates from high ($\lambda_{1}=0.4,\lambda_{21}=0.4$) to low ($\lambda_{1}=0.1,\lambda_{21}=0.1$) decreases SU2 packets’ average delay. This is because in overlay mode, the increase of high-priority user arrival rates reduces the opportunity of SU2 packets occupying the channel. This leads to SU2 packets’ waiting time in the buffer being longer. Then, average SU2 packet delay increases. In hybrid mode, lower high-priority user arrival rates increase the opportunity of SU2 packets transmitted with a high rate. This reduces the average transmission time and delay of SU2 packets. For underlay mode, the change of the high-priority user arrival rates has no effect on the average delay of SU2 packets. The reason is that, in underlay mode, SU2 packet transmission is constrained by a limited rate; thus, average SU2 packet delay is not affected by the change of high-priority user arrival rates. At the same time, we could find that, in the same condition, overlay mode had the lowest SU2 average delay, hybrid mode had middle SU2 average delay and the underlay model had the highest average delay. This may because SU2 packets’ transmission is interrupted by high-priority users in overlay access mode. When a high-priority user arrives, SU2 packets in the channels in overlay access mode leave the system earlier than those in hybrid access mode. This leads to the average delay in overlay access mode being lower than that in hybrid access mode. Compared to underlay access mode, the transmitting SU2 packets could dynamically switch between high and low transmission rates in hybrid access mode instead of always maintaining a low transmission rate in underlay access mode. Therefore, SU2 packets’ average transmission time in hybrid access mode was lower than that in underlay access mode. This means that the SU2 packets’ average delay in hybrid access mode was lower.

**Figure 7:**
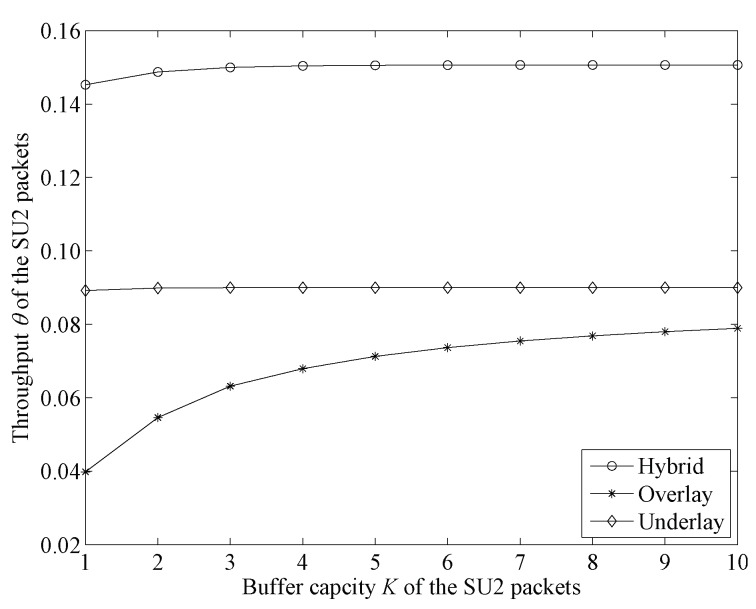
Throughput-change-trend comparison between three access modes with higher arrival rates for PU and SU1 packets ($\lambda_{1}=0.4,\lambda_{21}=0.4,M=3,\lambda_{22}=0.4,\mu_{H}=0.15,\mu_{L}=0.03,\mu_{1}=\mu_{21}=M\left(\mu_{H}-\mu_{L}\right)$).

Figure 7 shows the change trends of throughput in the three modes with higher arrival rates for PU and SU1 packets.

From Figure 7 we can see that, in the underlay and hybrid modes, the increase of buffer capacity had small impact on the throughput of SU2. This is because in these two modes, SU2 packet transmission is not interrupted by high-priority users. SU2 packet throughput is mainly influenced by transmission rate. In a low transmission rate, the buffer is very easily filled up. There are more SU2 packets waiting for transmission in the buffer and continuing to increase the buffer capacity has little effect on SU2 packet throughput. In overlay mode, the increase in buffer capacity has a significant impact on the increase of SU2 throughput when the buffer capacity is not very high. This is because more transmitting SU2 packets are interrupted by newly arrived high-priority user packets when we set higher arrival rates for high-priority users. Then, as buffer capacity increases, more SU2 packets can enter the system and the possibility for an SU2 packet being blocked decreases. This means that the possibility of an SU2 packet being transmitted is higher and the throughput of SU2 packets is increased. We also find that as the buffer capacity continues to grow, the increasing trend of SU2 packet throughput becomes less obvious. This is because the number of SU2 packets in the buffer may become saturated when the buffer capacity is increased to a certain degree.

Comparing these three modes, we could find that the proposed hybrid mode had the highest SU2 throughput and overlay mode had the lowest. This is because in the cases of hybrid and underlay access modes, the feature of the transmission of SU2 packets not being interrupted could guarantee transmission stability. In overlay mode, however, the transmitting SU2 packets are frequently interrupted, which results in smaller throughput.

**Figure 8:**
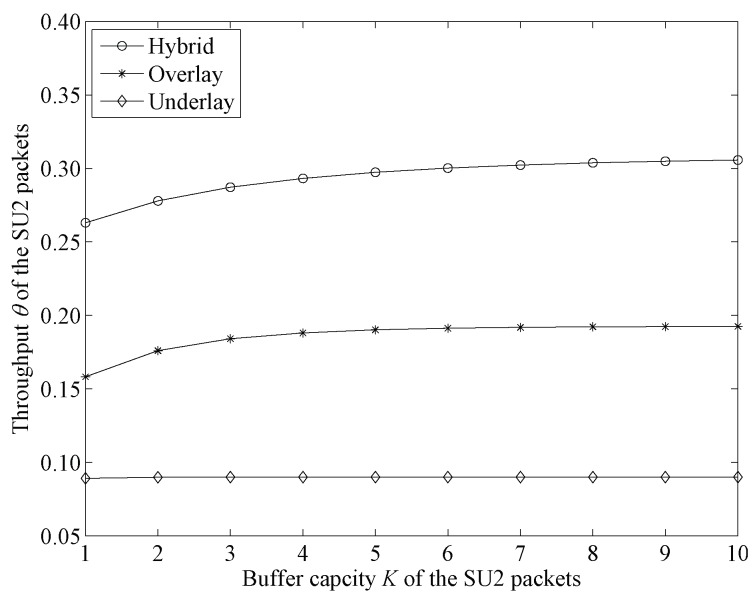
Throughput-change-trend comparison between three access modes with lower arrival rates for PU and SU1 packets ($\lambda_{1}=0.1,\lambda_{21}=0.1,M=3,\lambda_{22}=0.4,\mu_{H}=0.15,\mu_{L}=0.03,\mu_{1}=\mu_{21}=M\left(\mu_{H}-\mu_{L}\right)$).

Figure 8 shows throughput change trends in the three modes with lower arrival rates for PU and SU1 packets.

From Figure 8 we can see that, in underlay mode, the change line was exactly the same as the change line in Figure 7. This is because high-priority users had no effect on SU2 in this mode. In overlay and hybrid modes, the throughput of SU2 packets had obvious improvement compared with Figure 7. This is because in these two modes, lower arrival rates of high-priority users allow channels to have more time in an idle state, which makes SU2 packets have more opportunities to occupy the channels at a high transmission rate. Moreover, we compared these three modes and found that hybrid mode had the highest SU2 throughput, underlay mode had the lowest SU2 throughput and the SU2 throughput of overlay mode was in the middle. A difference from Figure 7 is that SU2 packet throughput in underlay mode was smaller than that in overlay mode. This indicates that, when channels have more time in idle state, overlay mode achieves better performance than underlay mode.

From Figure 6, Figure 7 and Figure 8 we can see that, compared with the traditional overlay and underlay modes, the proposed hybrid mode could always realize the highest SU2 throughput. The reasons are as follows. We noted that the performance of SU2 packets is not influenced by high-priority users and transmitted with a lower rate in underlay mode, whereas in overlay mode, SU2 packets could only be transmitted while the channel is not occupied. This means the performance of SU2 packets could be dramatically influenced by the arrival rates of high priority-users in overlay mode. We concluded that neither overlay nor underlay mode is ideal for SU2 packets, while the hybrid mode proposed in this paper inherited the transmission stability of underlay mode when the channel is in a busy state and the higher transmission rate of overlay mode when the channel is in an idle state. Therefore, the proposed hybrid mode realized the highest SU2 packet throughput.

## 5. Conclusions

In this paper, in order to enhance SU2 packet transmission stability and efficiency, we proposed a hybrid spectrum access mode with a channel bonding mechanism in CRNs with classified SUs. By establishing and analyzing a Markov chain model, we gave the system state-transition probability matrix and derived the expressions of some important SU2 packet performance measures, such as blocking rate, throughput and average delay. We also showed the change trends of SU2 packet average delay and throughput with numerical results. From these results we found that, for SU2 packets, an increase in buffer capacity increased throughput and average delay at the same time. Moreover, by comparing the numerical results of the proposed hybrid mode with the traditional overlay and underlay modes, we found that the throughput of SU2 packets could be significantly improved in our proposed hybrid spectrum access mode.

In the channel bonding scheme considered in this paper, we only assumed two cases: *M* channel bonding and no channel bonding. As future work, we are interested in analyzing an adaptive bond-size selection scheme in CRNs. In this paper, possible spectrum-sensing consumption was not considered. Therefore, spectrum-sensing consumption could also be an interest topic for our future research.
